# Age related prevalence of hand osteoarthritis diagnosed by photography (HOASCORE)

**DOI:** 10.1186/s12891-017-1870-0

**Published:** 2017-12-02

**Authors:** Helgi Jonsson

**Affiliations:** Department of Rheumatology, Landspitalinn University Hospital, University of Iceland, Fossvogur IS 108, Reykjavik, Iceland

**Keywords:** Hand osteoarthritis, Diagnosis, Photography, Epidemiology

## Abstract

**Background:**

Hand photography has been used in a number of studies to determine the presence and severity of hand osteoarthritis (HOA). The aim of this study was to present age and gender specific prevalences of HOA diagnosed by this method.

**Methods:**

Six thousand three hundred forty three photographs (from 3676 females and 2667 males aged 40–96) were scored for hand osteoarthritis by a 0–3 grade (0 = no evidence of OA, 1 = possible OA, 2 = definite OA and 3 = severe OA) for each of the three main sites, distal interphalangeal joints (DIP), proximal interphalangeal joints (PIP) and thumb base (CMC1). An aggregate score of 0–9 was thus obtained (HOASCORE) to reflect the severity of HOA in each case.

**Results:**

DIP joints were most commonly affected, followed by the thumb base and the PIP joints. Having definite DIP joint OA starts at a younger age compared with the other two sites, and there is a marked female preponderance in the age groups from 55 to 69, but after 70 the gender differences are less marked and the prevalence is fairly stable. PIP joint prevalence also indicates a female preponderance from 60 to 79. Thumb base OA has a more marked female preponderance and a rising prevalence thoughout life. The prevalence of individuals with no evidence of photographic OA (HOASCORE = 0) drops from 88% to 57% between the age categories 40–49 and 50–54 and decreased to 33% in the 70–74 age group with a slower decline after that age.

DIP and PIP prevalence were strongly associated with each other with an OR of 16.6(12.8–21.5),*p* < 0.001 of having definite OA at the other site. This was less marked for the thumb base with an OR of 2.2(1.8–2.7, *p* < 0.001), and 2.7(2.0–3.5, *p* < 0.001) of having definite DIP or PIP HOA respectively.

**Conclusions:**

The prevalence of hand OA in DIP, PIP and thumb base joints obtained by the photographic HOASCORE method is higher in women and increases after the age of fifty. These results are in line with those obtained by clinical examination and radiography. The advantage of the method lies in easy applicability and low cost**.**

**Electronic supplementary material:**

The online version of this article (10.1186/s12891-017-1870-0) contains supplementary material, which is available to authorized users.

## Background

Hand osteoarthritis (HOA) is a common condition that is associated with pain and disability [1, 2]. It is also a possible marker of the systemic nature of osteoarthritis, having associations with osteoarthritis at other sites [3, 4] and with atherosclerosis [5, 6].

The imaging of hand osteoarthritis is problematic. Radiography constitutes the gold standard for diagnosis, but shows limited associations with pain and function [7, 8]. Other methods such as magnetic resonance imaging (MRI), ultrasound and isotope scans may be more dynamic and informative with regard to individual joints and disease activity [9–11].

In a previous publication, our group presented a study standardizing the use of hand photographs for the diagnosis and severity of HOA in the elderly, using clinical examination and radiography as reference [8]. The photographic method was in most aspects comparable to the other methods in relation to pain and disability. Subsequent studies have indicated that this method of photographic scoring system is reliable and also a good indicator of hand OA in a younger population and offers a feasible alternative to physical examination and radiography [12].

In the current study, photographic data from two separate studies of population based participants 40 years and older are presented. The aim was to establish age related references for the prevalence of photographic hand osteoarthritis for future studies.

## Methods

High quality hand photographs from two population based studies were available for the assessment of age related prevalences. The AGES-Reykjavik study is a population based study of aging in elderly Icelanders (age 67+, *n* = 5170) [13]. Photographs from this study were initially used to standardize the reading of hand OA scores and compared to readings from radiographs and clinical examination. The second study „The Effect of CNV on the Genome “was a population based study of control subjects for neuropsychiatric CNV carriers in Iceland [14]. In this study a younger population based sample of individuals was recruited and hand photographs from participants 40 years old or older were analysed (*n* = 1173). The total number of photographs was thus 6343 (3676 females and 2667 males). Photographs in both studies were taken with high quality digital cameras mounted on a tripod with a fixed distance. Photographs were not scored joint by joint, but by an abbreviated score (HOASCORE). Each of the three joint sites, distal interphalangeal joints (DIP), proximal interphalangeal joints (PIP) and thumb base were scored for HOA by the author as previously described on a 0–3 scale for each (0 = unaffected, 1 = possible hand OA, 2 = definite hand OA and 3 severe hand OA. By this method, the emphasis is on severity in each joint group (DIP, PIP, thumb base) with additional considerations for symmetry and typical joints. For the DIP joints, definite nodal OA on one side, or bilateral suspected OA (scores of 1) were classified as 1 (some evidence of HOA). Bilateral definite nodal OA was required for a score of 2 (definite HOA) and bilateral definite OA plus one or more severely affected joints were required for a global score of 3 (severe HOA) at each site. For the PIP joints affection of more than 1 joint was required for a score of 2 (definite OA), but for the thumb base, unilateral severe involvement was sufficient for severe OA classification. [8]. An aggregate score of 0–9 was thus obtained. A score of 4 or more was chosen to reflect severe hand OA in accordance with previous studies [8, 12] A large number of reference photographs for all joint groups are available in the original article and a sample photograph from the original article is available in a Additional file 1. The HOASCORE readings were analysed in five year age categories 40–44 (*n* = 208), 45–49 (*n* = 238), 50–55 (*n* = 262), 55–59 (*n* = 290), 60–64 (*n* = 146), 65–69 (*n* = 556), 70–74 (*n* = 1555), 75–79 (*n* = 1505), 80–84 (*n* = 1167) and 85+ (*n* = 416).

Statistics were calculated using SPSS version 22. Chi-square and the Mantel Haenszel odds ratio estimate was used to calculate gender prevalence differences and the likelihood of having definite OA at a second site if participants had definite OA at one site.

## Results

**Fig. 1:**
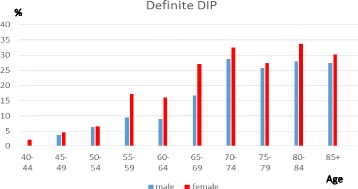
The prevalence of definite DIP joint OA (HOASCORE ≥ 2) in relation to age

**Fig. 2:**
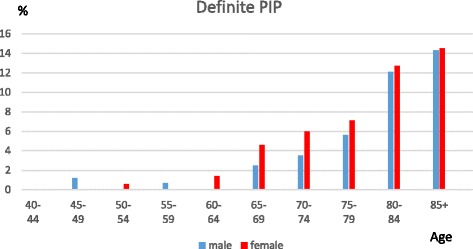
The prevalence of definite PIP joint OA (HOASCORE ≥ 2) in relation to age

**Fig. 3:**
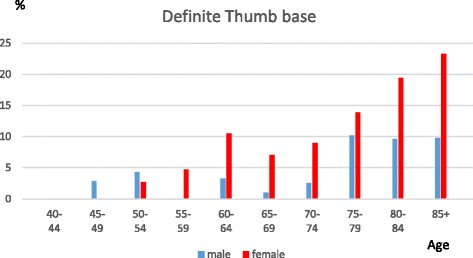
The prevalence of definite thumb base joint OA (HOASCORE ≥ 2) in relation to age

The age dependent prevalence of definite hand OA and the three main joint sites, DIP, PIP and the thumb base are presented in Figs 1-3. DIP joint OA (Fig. 1) starts at a younger age compared with the other two sites, and there is a marked female preponderance in the age groups from 55 to 69 (f143/740 vs m60/514;OR 1.8(1.3–1.5),*p* < 0.001), but after 70 the gender differences are less marked and the prevalence seems to stabilize after that age. For visual clarity reasons, standard errors of mean are only shown in a Additional file 2.

PIP joint OA is much less common than DIP joint OA on photographs. It starts at an older age but shows a similar pattern with a higher female preponderance in the sixties and seventies (f 129/2134 vs m68/1633; OR 1.5(1.1–2.0),*p* = 0.01) followed by fairly equal gender prevalence in the eighties (Fig. 2).

Thumb base OA prevalence is characterized by a more marked female preponderance throughout. Before 75 years of age it is much more prevalent in females, (f189/926 vs m46/657; OR 3.4(2.4–4.8), *p* < 0.001) but the prevalence increases in males after 75. Female prevalence continues to rise thoughout life (Fig. 3).

**Fig. 4:**
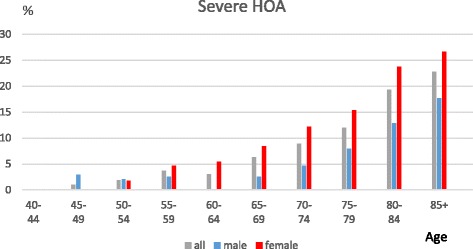
The prevalence of severe hand OA (HOASCORE ≥ 4) in relation to age

Figure 4 illustrates the age related prevalence of severe photographic hand OA, using an aggregate score of 4 or more to denote severe hand OA. It shows a marked gender difference, after the age of 55 (f497/3105 vs m185/2312; OR 2.2(1.8–2.6), *p* < 0.001) and a rising prevalence throughout life.

**Fig. 5:**
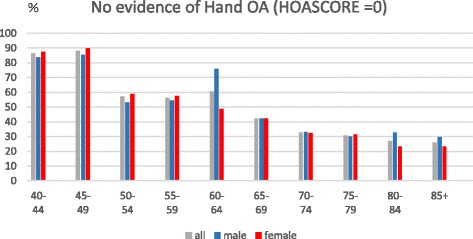
The prevalenc of having no evidence of hand osteoarthritis (HOASCORE = 0) in relation to age

The age related prevalence of those with no evidence of photographic hand OA (HOASCORE = 0) is shown in Fig. 5. There is a marked drop in the prevalence from 88% to 57% between the 45–49 and 50–55 age categories followed by age related decrease at a slower rate. Gender prevalences are similar.

**Fig. 6:**
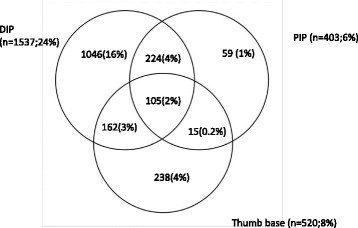
A diagram showing the overlap between having definite OA (HOASCORE ≥ 2) at any or all of the three sites, DIP, PIP and thumb base. Percentages refer to the whole study population (*n* = 6343)

Figure 6 illustrates the relationship between the three joint sites. The DIP and PIP sites are strongly associated and a definite HOA classification at either site meant an OR of 16.6(12.8–21.5),*p* < 0.001, of having HOA at the other site. The thumb base site is less strongly associated with having DIP (OR 2.2(1.8–2.7, *p* < 0.001) or PIP (OR 2.7(2.0–3.5, *p* < 0.001)). 105 (1.7%) individuals had definite OA classification at all three sites, constituting the most definite cases of generalized hand OA.

## Discussion

In this study, 6343 high quality hand photographs were scored with regard to hand osteoarthritis at the three main HOA sites, the DIP joints, the PIP joints and the thumb base. The aim of the study was to produce age-related reference data for photograpically diagnosed hand OA.

In our original study we compared 380 photographs, hand x-rays and clinical examination scores, showing reasonable correlations between the three metods for joint by joint scoring. The three methods also had comparable predictions for pain. In addition, the simplified HOASCORE showed a high degree of correlation with aggregate scores of both radiology and clinical examination [8]. These findings have been corraborated in a younger symptomatic population from the UK [12]. The age related prevalences obtained by photograph scoring in the current study show age related patterns that are comparable to clinical and radiographic studies, such as the marked female preponderance in the postmenopausal age groups and in the thumb base. Similarly, the stronger relationship between the presence of DIP and PIP OA compared with CMC1 has been described [15]. Of course, the scoring is quite different, with the HOASCORE scoring only joint sites and thus intended to give a general idea of the presence and severity of hand OA at the three sites in a given individual. It will never replace radiography or other methods which give more exact anatomic images of the joints themselves. The idea of developing a photographic scoring system for hand OA was not to improve the imaging of single joints with regard to hand OA, but rather to develop a simple and inexpensive method for assessing the burden of hand OA. In many situations such information can be useful in studies of associations with other aspects of OA and it’s systemic features. The distinct associations that have since been observed between photographic scores and the need for knee or hip joint replacements and systemic features such as atherosclerosis must be considered as indications of success for the method [3, 5].

Of course the limitations are many. In essence the HOASCORE method sacrifices anatomical detail for ease of use in effort to make it suitable for large studies. Important subsets such as erosive hand osteoarthritis cannot be diagnosed by photographs due to the non-visioning of the joint itself. The majority of those will simply be scored as severe OA (unpublished data). Other limitations include technical photographic problems such as shadows, sleeves or jewellery obscuring the thumb base and a certain sensitivity to thumb positioning on the photographs. Also the method seems to underestimate PIP joint involvement compared with radiographs and all scores in obese people as previously indicated [8]. In addition the method may have a limited sensitivity to change, at least over short periods of time. In a five year followup study of an AGES-Reykjavik Study subset (*n* = 143, mean age at second photo 79.5) we observed little progress in finger joints scores, but side by side comparisons revealed occasional worsening, most often in the thumb base [16]. Looking at the prevalence figures in Figs. 1 and 2, these results are now easier to understand since the prevalence of DIP and PIP joint OA by HOASCORE stays relatively constant at this age. Longitudinal studies aimed at measuring progress of HOASCORE in younger populations are now under way.

## Conclusions

The age and gender specific prevalences obtained by the photographic HOASCORE method of diagnosing hand osteoarthritis are consistent with a prevalence pattern where the three joint sites show variable increases in prevalence depending on age and gender, particularly after the age of fifty. The results also show similarities to those obtained by clinical examination and other imaging methods. Photographic scoring of hand OA appears to be a useful method in settings where more exact anatomic imaging is not a primary objective. It is much cheaper and easier to apply than all other imaging methods and has been shown to give relevant information about the burden of hand OA.

## Additional files

Additional file 1: An example of the use of the abbreviated photographic scoring system for hand OA. (DOCX 64 kb)

Additional file 2: Standard errors of mean. (XLSX 11 kb)

Additional file 3: Funding and acknowledgements for the original studies. (DOCX 11 kb)

## Abbreviations

CMC1: First carpometacarpal joint/thumb base
DIP: Distal interphalangeal joint
HOA: Hand osteoarthritis
HOASCORE: A photographic scoring system based on an aggregate severity score for each of the three joint sites DIP, PIP and thumb base.
MRI: Magnetic resonance imaging
PIP: Proximal interphalangeal joint
