# Supplementary material for: Age related prevalence of hand osteoarthritis diagnosed by photography (HOASCORE)
Source: BMC Musculoskelet Disord. 2017 Dec 2;18:508. doi: 10.1186/s12891-017-1870-0 (PMC5712087; doi:10.1186/s12891-017-1870-0)
Supplement: Supplementary file 1 — An example of the use of the abbreviated photographic scoring system for hand OA. (DOCX 64 kb) [file 12891_2017_1870_MOESM1_ESM.docx]

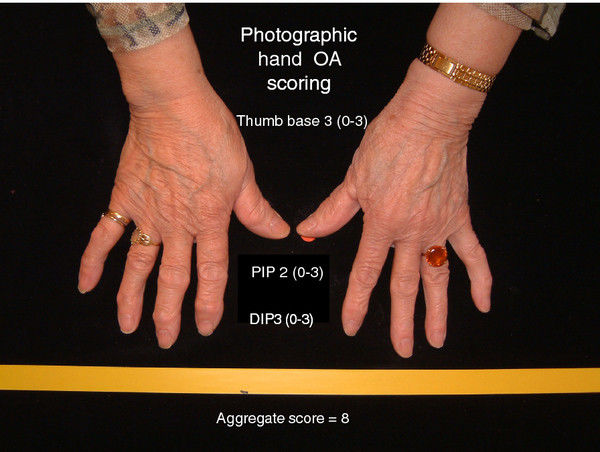


Figure 7

**An example of the use of the abbreviated photographic scoring system for hand OA**. Aggregate scores of ≥ 4 were assigned a score of 4, the most severe grading (HOASCORE).

Figure and text origin: [Jonsson H](https://www.ncbi.nlm.nih.gov/pubmed/?term=Jonsson%20H%5BAuthor%5D&cauthor=true&cauthor_uid=22340303), [Helgadottir GP](https://www.ncbi.nlm.nih.gov/pubmed/?term=Helgadottir%20GP%5BAuthor%5D&cauthor=true&cauthor_uid=22340303), [Aspelund T](https://www.ncbi.nlm.nih.gov/pubmed/?term=Aspelund%20T%5BAuthor%5D&cauthor=true&cauthor_uid=22340303), [Sverrisdottir JE](https://www.ncbi.nlm.nih.gov/pubmed/?term=Sverrisdottir%20JE%5BAuthor%5D&cauthor=true&cauthor_uid=22340303), [Eiriksdottir G](https://www.ncbi.nlm.nih.gov/pubmed/?term=Eiriksdottir%20G%5BAuthor%5D&cauthor=true&cauthor_uid=22340303), [Sigurdsson S](https://www.ncbi.nlm.nih.gov/pubmed/?term=Sigurdsson%20S%5BAuthor%5D&cauthor=true&cauthor_uid=22340303) et al. The use of digital photographs for the diagnosis of hand osteoarthritis: the AGES-Reykjavik study. [BMC Musculoskelet Disord.](https://www.ncbi.nlm.nih.gov/pubmed/22340303) 2012 16;13:20.
