# Supplementary material for: Age related prevalence of hand osteoarthritis diagnosed by photography (HOASCORE)
Source: BMC Musculoskelet Disord. 2017 Dec 2;18:508. doi: 10.1186/s12891-017-1870-0 (PMC5712087; doi:10.1186/s12891-017-1870-0)
Supplement: Supplementary file 3 — Funding and acknowledgements for the original studies. (DOCX 11 kb) [file 12891_2017_1870_MOESM3_ESM.docx]

**Acknowledgements**

*The Age, Gene/Environment Susceptibility Reykjavik Study (AGES-Reykjavik)* has been funded by NIH contract N01-AG-12100, the NIA Intramural Research Program, Hjartavernd (the Icelandic Heart Association), and the Althingi (the Icelandic Parliament), the Icelandic Osteoarthritis Fund and the University of Iceland Research Fund. The Effect of CNV on the Genome Study was sponsored by Decode Genetics. All data are results of the authors readings from photographs from these two studies.
